# Profiling and Characterization of Localized Cytokine Response in Congenital Cleft Affected Lip Tissue

**DOI:** 10.3390/life11060556

**Published:** 2021-06-13

**Authors:** Sophie Charlotte Reiser, Jonas Tellermann, Ilze Akota, Māra Pilmane

**Affiliations:** 1 Institute of Anatomy and Anthropology, Riga Stradins University, Kronvalda Boulevard 9, LV-1010 Riga, Latvia; 024340@rsu.edu.lv (J.T.); mara.pilmane@rsu.lv (M.P.); 2 Institute of Stomatology, Riga Stradins University, Dzirciema Street 20, LV-1007 Riga, Latvia; ilze.akota@rsu.lv

**Keywords:** immunohistochemistry, cytokines, children, cleft lip

## Abstract

(1) Background: Despite cleft lips and palates belonging to the most common orofacial congenital anomalies, their morphopathogenesis is not yet fully understood. The study aimed to determine the distribution and relation of cytokines interferon-γ (IFN-γ), tumor necrosis factor-alpha (TNF-α), interleukin (IL)-2, IL-7, IL-12, and IL-13 in the cleft affected mucosa of the lip. (2) Materials and Methods: Twenty cleft lip (CL) mucosal samples and seven control tissues of oral cavity mucosa were included in the study. Specimen were obtained during reconstruction surgeries and processed by hematoxylin and eosin staining and immunohistochemistry for IFN-γ, TNF-α, IL-2, IL-7, IL-12, and IL-13. (3) Results: The distribution of cytokines was higher overall in the cleft affected epithelium compared to the connective tissue, with TNF-a, IL-2, and IL-12 displaying the highest number of immunopositive cells. With the exception of IL-2, CL specimen showed higher immunoreactivity. IFN-γ displayed only minor immunoreactivity, with no expression in the control epithelium. Correlation analysis was strongest between CL epithelial IL-13 and IFN-γ (z = 0.71, *p* < 0.0001). (4) Conclusions: The CLP affected epithelium displays high degrees of plasticity in expressing different cytokines, pointing towards the stimulation of a local adaptive immune response based on consistent inflammatory processes.

## 1. Introduction

Orofacial clefts encompass a large group of congenital deformations with structural defects in the oral cavity and the adjacent facial framework. Within this group, the most common anomalies are cleft lips and palates (CLP), either occurring in isolation, in combined form, or as a part of an array of malformations [1]. The prevalence of CLP differs based on ethnicity, race, geographic location, and socioeconomic status [2], with 1.55 cases per 1000 live births in Europe [3].

A multifactorial model has been proposed for the development of CLP, though recent studies examining concordance rate and heritability between monozygotic twins with CLP pointed towards a strong genetic component within the multifactorial pattern [2]. The majority of CLP patients are subject to the non-syndromic form, where cleft lip and/or palate occur in the absence of other congenital anomalies. The less common syndromic form is associated with other deformations primarily backtracked to chromosomal or monogenic syndromes [1]. 

CLP affected patients suffer notably from a high degree of morbidity including impaired feeding, speech and language development, combined with restricted facial growth. A multidisciplinary management approach including repeated surgical interventions is required to restore their function and to improve the child’s facial appearance [2].

Oral health in postnatal CLP patients has been considered the major factor influencing a successful surgical outcome. The oral health in the CLP group has previously been evaluated with regards to the presence and degree of tooth decay, dental plaques, and gingival inflammation [4]. Reports on the oral microflora in CLP subjects described a higher grade of gingivitis in CLP patients when compared to a healthy control group, despite no observed difference in the degree of bacterial colonization [5]. While significantly higher gingival inflammation is reported in multiple studies, a precise identification and quantification of tissue mediators has not been subject to extensive research yet. 

**Figure 1:**
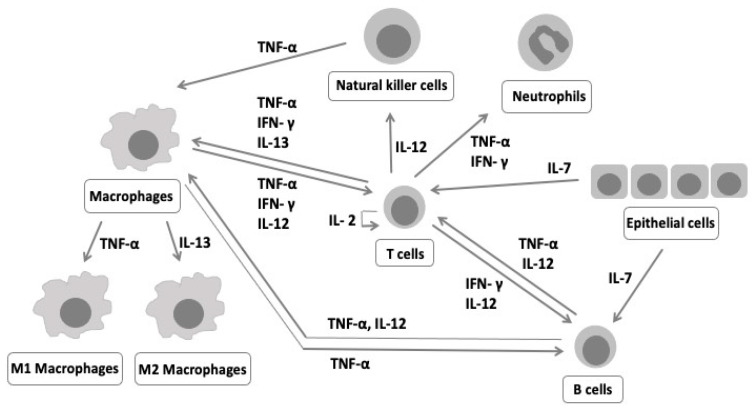
Cytokine interaction. Abbreviations: IL—interleukin, TNF-α—tumor necrosis factor-alpha, IFN-γ—interferon-gamma.

Interleukins and interferons have been described in the context of hyperplastic scar formation in the postoperative CLP patient [6] and in tissue remodeling during the chronic inflammatory state of gingivitis [7], making them of particular interest in postnatal CLP morphology. The interplay of the various cytokines and their correspondent immunoregulatory cells [8,9,10] can be visualized in a simplified form in Figure 1.

A plethora of cytokines has been implicated in the development and progression of oral cavity diseases, among them tumor necrosis factor-alpha (TNF-α). The pleotropic cytokine is secreted by an array of cells, namely macrophages, T cells, Langerhans cells, mast cells and keratinocytes [11]. A wide range of biological effects has been attributed to TNF-α, among them the inhibition of proliferation and stimulation of differentiation of keratinocytes. With the ability of keratinocytes to secrete TNF-α, research in the past decade pointed towards TNF-α as a key regulatory factor in the pathogenesis of oral lichen planus and periodontitis [12]. Elevated levels were depicted in local lesions of these diseases, contrary to the normal mucosa where the cytokine was nearly undetectable [13]. Along with oral inflammatory pathologies, the increased secretion of TNF-α by keratinocytes has also been reported in the state of dysplastic and neoplastic oral lesions [14]. Apart from tissue remodeling, increased serum levels of TNF-a have been associated with the development and progression of neuropathic pain, adding towards the considerable morbidity of cleft affected patients [15].

Interleukin (IL)-2, primarily produced by T helper-1 (Th-1) cells, is commonly known for the induction of proliferation and differentiation of T and B cells. Furthermore, the cytokine is involved in the activation of both natural killer (NK) cells and monocytes [16]. While oral mucosa keratinocytes demonstrate the presence of most pro-inflammatory cytokines (IL-1, IL-6, IL-8, TNF-α) even in a healthy state, IL-2 is not readily expressed in the normal mucosa [17,18]. Elevated IL-2 levels, however, were detected in patients with a history of recurrent aphthous stomatitis lesions. These findings were coherent with the cell mediated immune response provoked by IL-2. It should be noted that the same study, proving increased secretion of IL-2 in recurrent aphthous lesions, observed higher resting levels of previously mentioned pro-inflammatory cytokines and IL-2 in lesioned and non-lesioned mucosa of the affected patients compared to healthy subjects [19].

IL-7 is mainly produced by stromal cells in lymphoid organs and bone marrow, as well as keratinocytes and epithelial cells [20]. Like IL-2, it drives the development of T and B cells and is involved in the survival of naive and memory T cells. Besides its role in lymph node organogenesis, IL-7 and its receptor chains are expressed on lymphatic endothelial cells in dermal lymphatics. During acute and chronic skin inflammation, mice with an endothelial-specific deletion of IL-7 receptor alpha chain developed more edema compared to controls due to impaired lymphatic drainage [21]. An association between elevated IL-7 levels and Sjögren syndrome was observed in mice. Positively regulated by interferon-gamma (IFN-γ) producing Th-1 and CD8+ T cells in the salivary glands, IL-7 enhanced the expression of CXCR3 ligands in a T cell and IFN-γ dependent fashion and increased the level of TNF-α, facilitating tissue destruction and inflammation [22]. Sjögren’s syndrome is a chronic inflammatory autoimmune mediated condition of predominantly lacrimal glands with the histopathologic hallmark feature of localized lymphomononuclear infiltration of exocrine glandular tissue [23].

First described in 1980 [24], IL-12 was assigned a crucial role in the regulation of the adaptive immune response, promoting naive T-cell differentiation into Th1 cells, as well as the activation of NK and T-cells through IL-12, which in turn stimulates the production of other cytokines, primarily IFN-γ and TNF-α [25]. Release of IL-12 has been implicated in the initiation and progression of oral mucosa inflammatory pathologies. Most prominent is the association between IL, gingivitis, and periodontitis. Increased levels of IL-12 in the gingival mucosa of periodontitis affected samples were related to the severity of inflammation and bone destruction in tissue [26]. Another study demonstrated a co-occurrence of both elevated IL-12 and TNF-α in local lesions of gingivitis and chronic periodontitis compared to periodontal healthy subjects [9].

The T cell-derived type 2 cytokine IL-13 has been described in immunosurveillance, regulation of cancer progression, and neovascularization. By binding to its two receptor subunits, IL-13 receptor subunit alpha-1 and IL-13 receptor subunit alpha-2 (IL-13Ra2), downstream signaling cascades (involved in cell proliferation), cytostatic effects, or neoplastic cell death, are stimulated [27]. IL-13Ra2 overexpression and high affinity binding of IL-13 were detected in human head and neck cancers [28]. In the chronic inflammatory condition of reticular oral lichen planus, IL-13 mRNA levels were increased compared to healthy control tissue specimens [29]. 

The pro-inflammatory cytokine IFN-γ is predominantly secreted by activated T cells and NK cells, influencing a variety of biological processes such as macrophage activation, antigen presentation enhancement, immunity mediation, and the regulation of Th-1/Th-2 balance [30]. In head and neck squamous cell cancers, an IFN-γ mediated increase in apoptosis, inhibition of cancer cell viability, and cell migration was observed [31]. The expression of IFN-γ induced chemokines by epidermal keratinocytes is enhanced in dermatoses like psoriasis and atopic dermatitis [32]. 

Due to the influence of cytokines in a variety of oral inflammatory pathologies, this research aimed to identify the extent of distribution and spatial relationship of pro-inflammatory and immunoregulatory cytokines in the mucosal tissue of the cleft lip (CL).

## 2. Materials and Methods

### 2.1. Study Participants 

The patient group included twenty cleft affected children, fourteen boys and six girls, in the age range from 3 to 18 months (Median = 4; IQR = 3–5). Two patients were affected with bilateral CLP, five with unilateral right-sided CLP and thirteen with unilateral left-sided CLP. Tissue samples of the lip were obtained during lip plastic reconstruction surgeries, correcting the state of the cleft in the CLP Centre at the Institute of Stomatology of Riga Stradin¸š University. Reconstructive surgery was the definitive and sole treatment for all included patients. The soft tissue control group (CG) encompassed seven samples, five female and two male samples. All tissue controls were obtained during hyperdentia correctional surgery from erupted teeth. Control tissues contained oral cavity mucosa with underlying connective tissue. There was no evidence of any other pathologies in the tissue control samples.

The study was independently reviewed and approved by the local Ethical Committee of Riga Stradin¸š University (2003; 2013; 2018), and written informed consent was obtained from all parents after the explanation of the nature of this study. The study was performed in accordance with the Declaration of Helsinki, 2000.

### 2.2. Hematoxylin & Eosin Stain (H&E) and Immunohistochemistry (IHC) 

Tissues were fixed for one day in a mixture of 2% formaldehyde and 0.2% picric acid in 0.1 M phosphate buffer (pH 7.2). Afterwards, tissues were rinsed in thyroid buffer containing 10% saccharose for 12 h. Samples were embedded into paraffin. Three micrometer thick sections were cut and stained with hematoxylin and eosin (H&E) for subsequent evaluation. 

The HiDef Detection HRP Polymer system (Cell MARQUE) was used for the detection of IFN-γ (code ab218426, rabbit polyclonal antibody, working dilution 1:500, Abcam, Cambridge, UK), TNF-α (code ab6671, rabbit polyclonal antibody, working dilution 1:200, Abcam, Cambridge, UK), IL-2 (code ab92381, rabbit monoclonal antibody, working dilution: 1:250, Abcam, Cambridge, UK), IL-7 (code orb13506, rabbit polyclonal antibody, working dilution 1:100, Biorbyt, St. Louis, MO, US), IL-12 (code ab10894, rabbit polyclonal antibody, working dilution 1:200, Biorbyt, St. Louis, MO, US). IL-13 (code ob10895, rabbit polyclonal antibody, working dilution 1:200, Biorbyt, St. Louis, MO, US), and anti-macrophage inflammatory protein 1 beta (MIP-1ß) (ab9675, working dilution 1:100, Abcam, Cambridge, UK).

### 2.3. Visualization and Statistical Analysis 

The specimens were analyzed using light microscopy with grading of immunoreactive structures in a semi-quantitative manner. A scale from “0” to “++++” was used for the evaluation of epithelium and connective tissue with the following definitions: “0“—no immunoreactive structures present in the visual field, “0/+”—occasional immunoreactive structures in the visual field, “+”—few immunoreactive structures in the visual field, “+/++”—few to moderate immunoreactive structures in the visual field, “++”—moderate immunoreactive structures in the visual field, “++/+++”—moderate to numerous immunoreactive structures in the visual field,“+++”—numerous immunoreactive structures in the visual field, “+++/++++”—numerous to abundant immunoreactive structures in the visual field, “++++”—abundant immunoreactive structures in the visual field [33]. Images of specimens were captured using a Leica DFC 450 digital camera. 

IBM SPSS version 22.0 was used for the statistical analysis of data. Descriptive analysis was performed, and nonparametric Mann–Whitney U test was utilized for the comparison of the two study groups. Spearman’s rank correlation coefficient (ρ) with (ρ) = 0.9–1 displaying very high positive correlation, (ρ) = 0.7–0.9 high positive correlation, (ρ) = 0.5–0.7 moderate positive correlation, (ρ) = 0.3–0.5 low positive correlation, and (ρ) = 0.0–0.3 negligible correlation was used [34]. Results with a *p*-value < 0.05 were considered statistically significant.

## 3. Results

**Figure 2:**
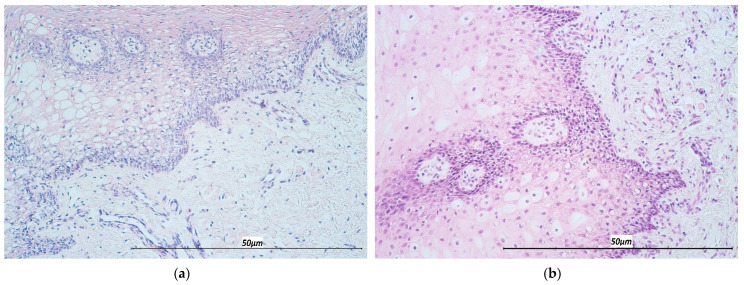
Hematoxylin & Eosin staining of lip mucosal tissue in two selected cases of cleft lip patients. (**a**) Note basal cell hyperplasia and vacuolization in the epithelium (200×). (**b**) Note the predominantly inflammatory cell infiltration in the subepithelial tissue folds (200×).

The analysis of routine stained CL specimens displayed the presence of stratified squamous epithelium and underlying connective tissue with varying degrees of chronic inflammatory cells. Varying degrees of vacuolization were observed in the epithelium, as well as basal cell hyperplasia and intraepithelial lymphocyte infiltration (Figure 2a,b).

### 3.1. Immunohistochemistry

**Figure 3:**
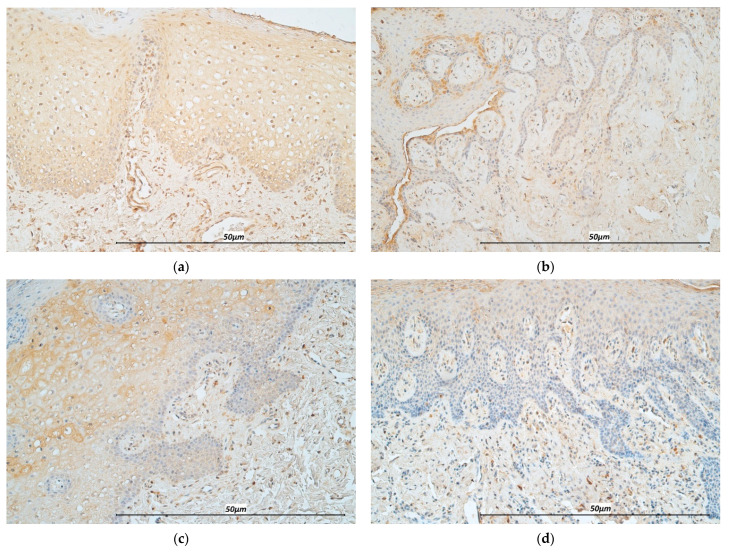
Immunohistochemistry micrographs of cleft affected tissue and controls. (**a**) Note numerous-abundant TNF-α immunoreactive cells in the lip epithelium of a cleft affected child (TNF-α, 200×). (**b**) Note the presence of few-moderate immunoreactive epitheliocytes in the control sample (TNF-α, 200×). (**c**) Note numerous IL-12 positive cells in the cleft affected connective tissue (IL-12, 200×). (**d**) Note moderate number of immunoreactive cells in the connective tissue of the control (IL-12, 200×).

TNF-α distribution, both in the epithelium and connective tissue, displayed a mean of moderate to numerous immunoreactive cells. Histopathological analysis of subepithelial stained cells showed primarily cells of chronic inflammatory origin, particularly macrophages, lymphocytes, and fibroblasts. TNF-α reactive cells in CG specimens were less prevalent in the epithelium and subepithelium (Figure 3a,b).

IL-12 in epitheliocytes and connective tissue cells was of comparable quantity in stained number of cells. Levels of IL-12 were decreased in the CG, where epithelium and underlying connective tissue displayed a mean of only few to moderate immunoreactive structures (Figure 3c,d).

**Figure 4:**
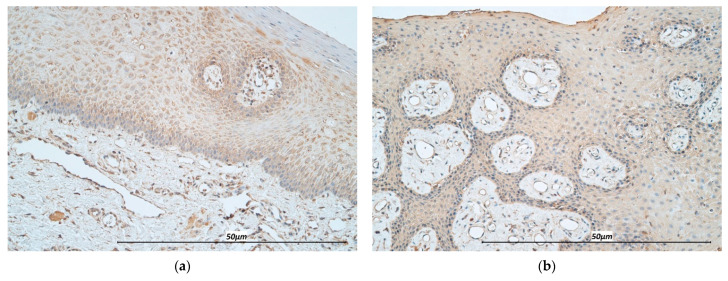
Immunohistochemistry micrographs of cleft affected tissue and controls. (**a**) Distribution of numerous IL-2 positive epitheliocytes and connective tissue cells in the cleft-affected lip mucosal tissue (IL-2, 200×). (**b**) Note abundant IL-2 positive cells in the control epithelium (IL-2, 200×).

A lower number of IL-2 immunoreactive cells was detected in the CL epithelium compared to the healthy tissue. In the connective tissue, positive structures for cytokine IL-2 were higher in the CL samples than in the CG (Figure 4a,b). 

**Figure 5:**
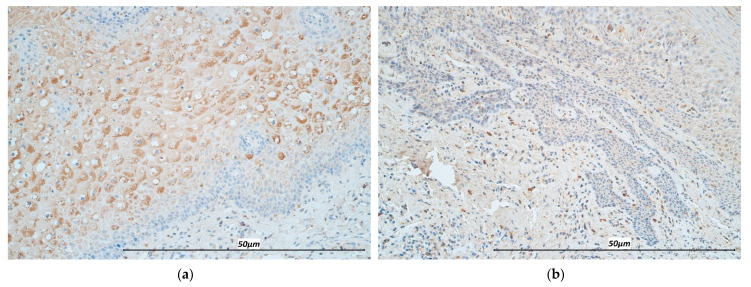
Immunohistochemistry micrographs of cleft affected tissue and controls. (**a**) Appearance of numerous IL-7 positive epitheliocytes and few connective tissue cells in cleft affected tissue (IL-7, 200×). (**b**) Note few-moderate IL-7 immunoreactive epitheliocytes and few connective tissue cells in the control (IL-7, 200×).

The distribution of IL-7 in the epithelium of CL specimens varied from occasional to numerous immunoreactive epithelial cells, whereas mainly few IL-7 positive cells were present in the connective tissue. Both in the epithelium and connective tissue of the CG, immunoreactive structures were less prevalent, ranging from few to few to moderate in the epithelium, and occasional to few to moderate in the subepithelium, respectively (Figure 5a,b). 

**Figure 6:**
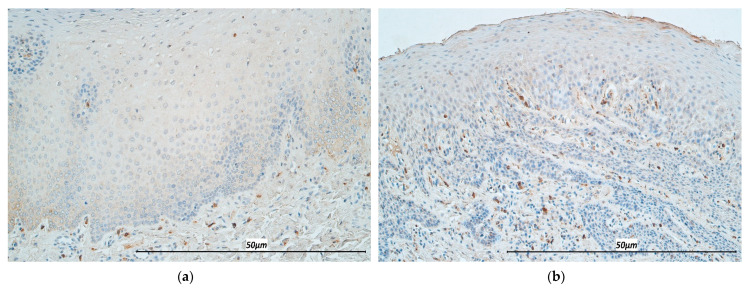
Immunohistochemistry micrographs of cleft affected tissue and controls. (**a**) Note few-moderate number of IL-13 positive connective tissue cells in the cleft tissue (IL-13, 200×). (**b**) Appearance of moderate number of IL-13 positive connective tissue cells in the control (IL-13, 200×).

IL-13 immunoreactive cells were detected in the range from few to numerous in the CL epithelium, and few to moderate in the CG epithelium. The cleft group and controls presented with a mean distribution of few to moderate IL-13 immunoreactive cells in the connective tissue (Figure 6a,b). 

**Figure 7:**
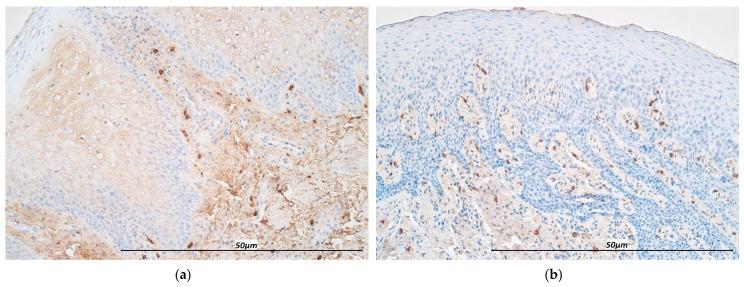
Immunohistochemistry micrographs of cleft affected tissue and controls. (**a**) IFN-γ displays moderate number of positive weakly stained epitheliocytes and connective tissue cells in a cleft affected tissue (IFN-γ, 200×). (**b**) Lack of IFN- γ immunoreactive cells in the epithelium, but a moderate number of them in the connective tissue of control (IFN-γ, 200×).

Three CL specimen displayed no IFN-γ immunoreactivity in the epithelium. The remaining 17 samples presented with occasional to numerous immunopositive epitheliocytes, contrasted by the immunonegativity of epithelial cells in all controls. Mean IFN-γ distribution in the connective tissue was few to moderate (+/++) both in the CL group and the control group (Figure 7a,b). 

Overall, analyzing the marker distribution in the CL specimen, IL-2 and IL-12 showed the highest mean number of immunoreactive cells both in the epithelium and connective tissue, while IFN-γ showed the lowest (Table 1).

**Figure 8:**
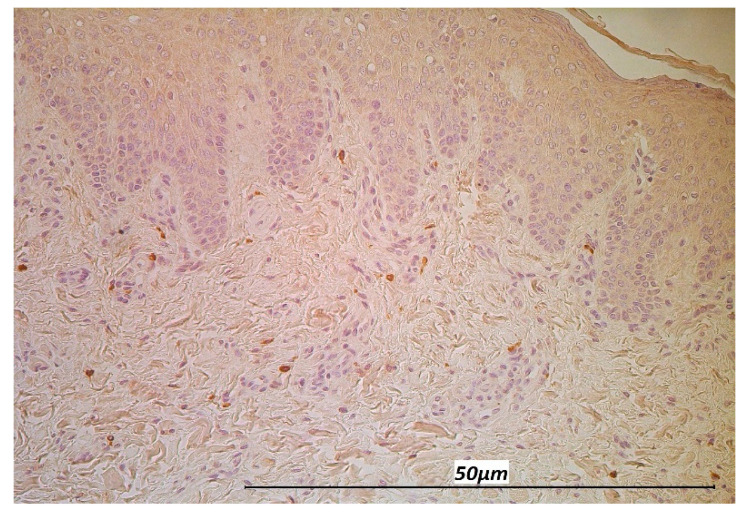
MIP-1ß immunoreactive macrophages located diffusely in the subepithelium of the CL tissue in a 6-month-old child (MIP-1ß, 200×).

Further immunohistological staining confirmed the presence of MIP-1ß positive macrophages in the inflammatory infiltrate of CL affected individuals (Figure 8).

Overall, no gender difference was observed in the cytokine expression or distribution in the cleft affected individuals.

### 3.2. Statistical Analysis

Correlation analysis of the CLP affected group (Table 2) displays a high positive correlation in the epithelium between IL-13 and IFN-γ. 

A moderate positive correlation was noted in the CL epithelium between IL-2 and TNF-α, IL-2 and IL-12, IL-2 and IL-13, IL-7 and IL-12, IL-12 and TNF-α, and IL-13 and IL-12, and in the connective tissue between IL-2 and IL-12 as well as between IL-13 and IL-12. Additionally, a moderate positive correlation was detected between the TNF-α distribution in the epithelium and the connective tissue.

A statistically significant difference between the marker distribution in the cleft affected group and the control group (Table 3) was observed with IL-2 in the epithelium, IL-7 in the epithelium, IL-12 in the epithelium and connective tissue, and IFN-γ in the epithelium.

## 4. Discussion

As an initial physical barrier, the oral mucosa provides a well-adapted defense to an array of environmental pressures and commensal and innocuous antigens without provoking a disproportionate inflammatory response [35]. Immunoregulatory response in our patients favored a prominent pattern of cytokines IL-7, IL-12, and IFN-γ predominantly in the cleft affected epithelium, seemingly indicating the specific role of those cytokines in the protective function. 

In the adaptive immune response, IFN-γ is predominantly secreted by Th1 cells [30]. No link between IFN-γ and local immune mucosal response in cleft mucosa has been established to this point. However, we speculate that the increased levels of IFN-γ in line with TNF-α are successors of a sustained activation of M1 macrophages, which by themselves induce the prototypic T cell mediated chronic inflammatory response that we observed in the cleft tissue samples. This observation is supported by the research of others [9]. An example of T cell mediated chronic inflammatory response is oral lichen planus (OLP) [36], in which infiltrating monocytes are recruited into oral mucosa developing a pro inflammatory M1 phenotype due to high levels of TNF-α and IFN-γ at the lesion site. IFN-γ primed M1 macrophages are considered a major determination in aiding progression of OLP through the activation of T cells, destruction of the basal membrane, and exacerbating mucosal erosions [37]. In agreement with our results, erosive OLP lesions showed a higher expression of IFN-γ in direct comparison to a healthy mucosa [29].

Elevated IL-12 expression in the cleft affected mucosa proved to be in accordance with our hypothesis of a predominantly T-cell mediated consistent inflammatory state. It is noteworthy that both the oral epithelium and the underlying lamina propria showed significantly higher degrees of IL-12 expression. Previous reports on the significance of chronic oral diseases were inconsistent. While Shaddox et al. [38,39] suggest a significant role of IL-12 in localized aggressive periodontitis in two studies, it contrasts the findings of others. However, IL-12 production remains essential in directing activated T-cell conversion to a Th-1 phenotype with a high IFN-γ secretion [40]. IL-12, a product of antigen presenting cells, and IFN-γ provide the central link in connecting the innate to the adaptive immune response.

We suggest that, considering the elevation of both cytokines IL-12 and IFN-γ, and given their association, that a profound interaction between T-cell and antigen presenting cells together with a raised activation of innate immune cells produces a persistent chronic inflammation in the cleft oral mucosa. Interestingly, an increasingly stimulated IL-12 production and in turn amplified Th-1 cell response, by antigen presenting cells, especially dendritic cells, has been reported in response to oral bacterial pathogens [41]. This falls in line with previous findings of a significant increase in commensal and potentially pathogenic microbial organisms in cleft affected neonates compared to their healthy counterparts [42].

Regarding the distribution of IL-7 in the cleft affected epithelium, our findings might indicate a possible association between epithelial proliferation and inflammation, as increased levels of immunoreactive epitheliocytes were seen in lip specimens. While the role of IL-7 in the context of the oral epithelium is not well elaborated yet, studies on chronic inflammatory diseases indicate a link between increased IL-7 levels and inflammatory states. Bikker et al. [43] observed increased IL-7 levels in the labial salivary glands of patients with primary Sjögren syndrome, as well as IL-7 induced cytokine production of TNF-α and IL-13. Apart from possible modulating effects in persistent inflammatory states, IL-7 is mainly recognized in the homeostasis of T cells [44]. We could not detect any observable differences between cleft affected samples and control samples in the mean IL-7 distribution in the subepithelium. Therefore, IL-7 does not seem to be specific to the oral subepithelial connective tissue of the cleft affected children.

Increased TNF-α and IL-12 CL connective tissue response may indicate a linkage and common function in the development of the local tissue immunity. Reports on the increased incidence of mucosal ulceration, gingivitis, caries and a general poor oral health in the cleft affected [45,46] gives reason to assume that the cleft pathology exposes its carrier in a higher degree to chronic inflammatory diseases of the oral mucosal lining. This assumption aligns with our findings of marked elevated TNF-α levels in the cleft group over the healthy comparison group. We suspect that the high levels of pro-inflammatory cytokine TNF-α and the accompanying inflammatory tissue response are the result of a yet unknown environmental trigger unique to the cleft epithelium. 

TNF-α is considered a main modulator in the biological response triad of inflammation, cell proliferation, and tissue remodeling [47]. Although not yet described in cleft pathology, an increased oral mucosal level of TNF-α has been outlined in a variety of chronic oral inflammatory conditions, most notably in periodontitis, in which TNF-α has been linked to ongoing soft and hard tissue destruction [48]. Consistent with a persistent inflammatory response in CLP mucosa, a report on the expression of inflammation related signaling molecules in healed oral mucosal scars did demonstrate TNF-α negativity in all their observed cases [49]. Furthermore, a transient enhancement of TNF-α mediated inflammatory response in the epithelium through synchronous upregulation of IL-1ß has been suggested through Saperstein et al. [50]. IL-1ß is considered to induce modification of TNF receptor expression and its subsequent shedding on the epithelial surface. IL-1 has previously been described as a predominant cytokine in cleft affected mucosa [51].

A dominant IL-12 response in inflammatory mucosal lesion points towards a high activation of pro-inflammatory M1 macrophage phenotype, whereas the M2 phenotype is associated with low IL-12 levels [52]. A prolonged M1 activity is tied to adverse effects on wound healing [53].

Interestingly, IL-2 displayed higher numbers in the epithelium of controls compared to the cleft group. IL-2 is one of the instrumental cytokines in priming and maintaining a cell mediated immune response. Our data showed consistent elevated levels of IL-2 over both tissue groups. Expression in epithelium proved dominant in healthy tissue, while subepithelial levels where higher in cleft affected tissue. Interestingly, preceding reports reported on elevated expression of IL-2 in lesions of predominantly T-cell mediated inflammatory diseases [29,54]. IL-2, primarily secreted by antigen primed Th-1 cells, remains the essential growth and differentiation factor for both subsets of Th-1 and Th-2 cells. We suspect that the IL-2 expression in cleft tissue reflects a Th1-cell driven, predominantly subepithelial, inflammatory reaction local to the oral mucosa. A positive correlational analysis for other markers (TNF-α, IL-12), favoring a Th1 directed tissue response, is in support of our theory. A local T-cell mediated inflammatory response has been previously described in oral lichenoid reactions. However, reports alternate between a Th-1 cell and Th-2 cell predominant pathogenetic role [29]. 

The main novelty of our work is the complex research on the tissue factors in the unique age group of children before and during the milk dentition age. However, we realize that an additional quantification of tissue markers by standardized laboratory measurements (e.g., ELISA), would be beneficial to the purely visual evaluation of immunohistochemistry stained samples. The use of additional techniques remained limited by the small sample size available. Furthermore, we acknowledge that the use of a hyperdentic control group might pose a limitation to an opposed completely healthy mucosal specimen. Ethical considerations, however, mandate the use of this relative control group. Due to data limitations, we were unable to differentiate between the syndromic and non-syndromic nature of the clefts.

## 5. Conclusions

The CLP affected epithelium shows high plasticity in expressing different cytokines. The increase of IL-7, IFN-γ, and IL-12 in the CLP epithelium suggests the stimulation of local adaptive immune responses based on consistent inflammatory processes. The decrease in epithelial IL-2 seems specific for the CLP affected epithelium, suggesting a possible decrease of immune cell proliferation and differentiation. IL-13 and TNF-α are not the most characteristic cytokines of CLP tissue, while the high positive correlation between IL-13 and IFN-γ in chronic inflammatory circumstances proves the possible stimulation of macrophages.

## Figures and Tables

**Table 1:** Distribution of immunoreactive cells in the epithelium and connective tissue of cleft lip affected specimens. Mean distribution of immunoreactive cells in control samples.

Cleft Affected Group												
	TNF-α		IL-12		IL-2		IL-13		IL-7		IFN-γ	
Subjects	EP	CT	EP	CT	EP	CT	EP	CT	EP	CT	EP	CT
1.	+++	++	+++	+++	+++	++	+++	++	++	0/+	++/+++	++
2.	+++	++	+++	+/++	+++	+	+++	+	+++	+	++/+++	+/++
3.	+/++	0/+	++/+++	0/+	+	0/+	0/+	0/+	++/+++	+	+/++	+
4.	++	++/+++	++/+++	++	+++	++	++/+++	+/++	++	+	++/+++	++
5.	++	+++/++++	+	+++	+/++	++/+++	0/+	+/++	+/++	+	0	++
6.	+++	+++	+++	+++	+++	+++	++/+++	+/++	+++	+/++	+/++	+/++
7.	+++	+++/++++	++	+/++	+++	++/+++	0	+/++	++	+/++	+/++	+/++
8.	+++	+++	+++	+++	+++	+++	++/+++	+/++	++	+	+	0/+
9.	+++	+++	+++	++/+++	+++	++/+++	+/++	+/++	+++	++	0	+/++
10.	++/+++	++/+++	+++	+++/++++	+++	+++	0/+	++/+++	0/+	0/+	0/+	0/+
11.	+/++	++	++/+++	++	+/++	+	0	+/++	++/+++	+	+	+/++
12.	+++	++	+++	+++	+++	+++	+++	+/++	+++	+	+++	+/++
13.	+	0/+	+/++	+/++	++	++	0	0/+	+/++	0/+	0	0/+
14.	++/+++	+++	++/+++	+++	++/+++	++/+++	+/++	++/+++	++	+	+	+/++
15.	+++/++++	+++	+++	++/+++	+++	+++	++	+/++	++/+++	+	++	+/++
16.	+++/++++	+++/++++	++++	+++	+++	+++	++/+++	++	+++	+	++/+++	++
17.	+/++	+/++	+++	+++	+++	+++	++/+++	++	++/+++	+	++/+++	++
18.	+	++	++/+++	++/+++	+++	+++	++	+/++	+++	+	++	+/++
19.	+/++	0/+	+++	++	+++	++/+++	++/+++	++	+++	+	+	++
20.	++/+++	++	++/+++	++	+++	+++	++/+++	++	++/+++	+/++	+/++	++
**Mean**	++/+++	++/+++	++/+++	++/+++	++/+++	++/+++	++	+/++	++/+++	+	+/++	+/++
Control Group												
**Mean**	++	+/++	+/++	+/++	+++/++++	++	+/++	+/++	+/++	+	0	+/++

Abbreviations: EP—epithelium, CT—connective tissue, IL—interleukin, TNF-α—tumor necrosis factor-alpha, IFN-γ—interferon-gamma, 0—no immunoreactive structures, 0/+—occasional immunoreactive structures, +—few immunoreactive structures, +/++—few to moderate immunoreactive structures, ++—moderate immunoreactive structures, ++/+++—moderate to numerous immunoreactive structures, +++—numerous immunoreactive structures, +++/++++—numerous to abundant immunoreactive structures, ++++—abundant immunoreactive structures.

**Table 2:** Correlation analysis of the cleft affected group displays positive correlations between different markers in the epithelium and connective tissue of lip specimens.

Strength of Correlation	Marker 1	Marker 2	ρ	*p*-Value
High positive correlation (ρ = 0.7–0.9)	IL-13 in EP	IFN-γ in EP	0.71	<0.0001
Moderate positive correlation (ρ = 0.5–0.7)	TNF-α in EP	TNF-α in CT	0.647	0.002
	IL-13 in EP	IL-12 in EP	0.646	0.002
	IL-2 in EP	IL-12 in EP	0.64	0.002
	IL-2 in EP	IL-13 in EP	0.637	0.003
	IL-2 in CT	IL-12 in CT	0.635	0.003
	IL-13 in CT	IL-12 in CT	0.628	0.003
	IL-12 in EP	TNF-α in EP	0.589	0.006
	IL-2 in EP	IL-2 in CT	0.577	0.008
	IL-7 in EP	IL-12 in EP	0.514	0.02
	IL-2 in EP	TNF-α in EP	0.512	0.021

Abbreviations: EP—epithelium, CT—connective tissue, IL—interleukin, TNF-α—tumor necrosis factor-alpha, IFN-γ—interferon-gamma.

**Table 3:** Statistically significant differences between the cleft lip and palate affected group and controls.

Marker	Mann–Whitney U	Z-Score	*p*-Value
IL-2 in EP	7.5	−3.3364	0.002
IL-7 in EP	9.0	−3.169	0.001
IL-12 in EP	8	−3.288	0.001
IL-12 in CT	19	−2.559	0.011
IFN-γ in EP	7.5	−2.959	0.02

Abbreviations: EP—epithelium, CT—connective tissue, IL—interleukin, IFN-γ—interferon-gamma.

## Data Availability

The data that support the findings of this study are available from the corresponding author upon reasonable request.
